# Relation Between Gender and Concomitant Medications With Erythropoietin-Treatment on Wound Healing in Burn Patients. Post Hoc Subgroup-Analysis of the Randomized, Placebo-Controlled Clinical Trial “EPO in Burns”

**DOI:** 10.3389/fphar.2022.812888

**Published:** 2022-07-01

**Authors:** Christina Irene Günter, Felicitas Paula Ilg, Alexander Hapfelmeier, Silvia Egert-Schwender, Wolfgang Jelkmann, Shibashish Giri, Augustinus Bader, Hans-Günter Machens, and EPO in Burns Study Group

**Affiliations:** ^1^ Clinic for Plastic Surgery and Hand Surgery, Klinikum rechts der Isar, Technical University Munich, Munich, Germany; ^2^ Institute of Medical Informatics, Statistics and Epidemiology, Klinikum rechts der Isar, Technical University Munich, Munich, Germany; ^3^ Müncher Studienzentrum, Klinikum rechts der Isar, Technical University Munich, Munich, Germany; ^4^ Institute for Physiology, University of Lübeck, Lübeck, Germany; ^5^ Institute for Cell Techniques and Applied Stem Cell Biology, University of Leipzig, Leipzig, Germany; ^6^ Clinic for Plastic and Hand Surgery, Klinikum rechts der Isar, Technical University Munich, Munich, Germany

**Keywords:** erythropoietin (EPO), burn injuries, gender, regenerative medicine, wound healing, randomized clinical trial

## Abstract

Burns are leading causes of mortality and morbidity, including prolonged hospitalization, disfigurement, and disability. Erythropoietin (EPO) is a well-known hormone causing erythropoiesis. However, EPO may play a role in healing acute and chronic wounds due to its anti-inflammatory and pro-regenerative effects. Therefore, the large, prospective, placebo-controlled, randomized, double-blind, multi-center clinical trial “EPO in Burns” was initiated to investigate the effects of EPO versus placebo treatment in severely burned patients. The primary endpoint of “EPO in Burns” was defined as the time elapsed until complete re-epithelialization of a defined split skin graft donor site. Additional analyses of post hoc defined subgroups were performed in view of the primary endpoint. The verum (n 45) and control (n 39) groups were compared with regard to the time it took for study wounds (a predefined split skin graft donor site) to reach the three stages of wound healing (re-epithelialization levels). In addition, the effects of gender (females n 18) and concomitant medications insulin (n 36), non-steroidal anti-inflammatory drugs (NSAIDs) (n 41), and vasopressor agents (n 43) were tested. Life tables were used to compare study groups (EPO vs. placebo) within subgroups. The Cox regression model was applied to evaluate interactions between the study drug (EPO) and concomitant medications for each re-epithelialization level. Using our post hoc defined subgroups, we observed a lower chance of wound healing for women compared to men (in terms of hazard ratio: hr^100%^: 5.984 [95%-CI: (0.805–44.490), *p* = 0.080]) in our study population, regardless of the study medication. In addition, results indicated an earlier onset of re-epithelialization in the first days of EPO treatment (EPO: 10% vs. Placebo: 3%). Moreover, the interpretation of the hazard ratio suggested EPO might have a positive, synergistic effect on early stages of re-epithelialization when combined with insulin [hr^50%^: 1.307 (*p* = 0.568); hr^75%^: 1,199 (*p* = 0.715)], as well as a stabilizing effect on critically ill patients [reduced need for vasopressors in the EPO group (EPO: 44% vs. Placebo 59%)]. However, additional high-quality data from clinical trials designed to address these endpoints are required to gain further insight into these effects.

## Introduction

Burn injuries are a leading cause of morbidity and mortality worldwide and remain a major reason for disability or personal and social impairments. If they survive, burn patients suffer from lifelong physiological, physical, and psychological disabilities (Church et al., 2006; Greenhalgh et al., 2007). With approximately one million adult patients worldwide per year requiring specialized medical treatment, major burns continue to be a leading cause of death and morbidity (World Health Organization, 2004; Lee et al., 2014; World Burn Foundation, 2020).

Losing the protective barrier of their skin puts burn patients at high risk. Typical manifestations of a severe burn injury are immunosuppression, hyper-metabolism, and complications such as wound infection and sepsis, with subsequent multi-organ failure and death (Dokter et al., 2015). Therefore, burns lead to the risk of delayed and poor recovery. For this reason, early wound closure is essential for the prognosis and rehabilitation of burn patients.

For 4 decades, millions of patients have received erythropoietin (EPO) as front-line therapy for several types of anemia, improving their prognoses and quality of life. Reports on non-hematopoietic effects of EPO in systemic application were first published around the turn of the millennium (Bany-Mohammed et al., 1996; Fatouros et al., 1999; Lamon and Russell, 2013; Alural et al., 2014). Cytoprotective effects of EPO have since been described in many studies on different animal models, organs, organ systems, and cells (Corwin et al., 2007; Osato et al., 2018), including skin and wound healing (Galeano et al., 2006; Bader et al., 2012; Giri et al., 2015). Moreover, due to its anti-inflammatory and anti-apoptotic effects, EPO was expected to play a promising role in healing and restoration (*restitutio ad integrum*) after trauma (Arcasoy, 2008; Brines and Cerami, 2008; Zhang et al., 2014), in addition to its erythropoietic action (Jelkmann and Jelkmann, 2013).

Hamed et al. describe improved wound healing after topical EPO applications, using various animal models (rats and pigs), in burn injuries and chronic wounds (Hamed et al., 2010; Hamed et al., 2014; Hamed et al., 2017).

In a large multi-center trial, Corvin et al. investigated the safety of EPO administration in poly-traumatized patients, demonstrating that the use of systemically applied EPO is safe and beneficial for these patients. Thromboembolic events could thus be reduced to ranges seen in poly-traumatized patients not receiving EPO when combined with appropriate anti-thrombotic therapy (Corwin et al., 2007). In our previous publication of “EPO in Burns,” we presented primary and secondary endpoint analysis data, including safety, regenerative effects, and efficacy of systemically applied (s.c.) EPO in split skin graft donor sites, as well as in burn and scald injuries, and severely burned patients (Gunter et al., 2018). However, the analysis of the primary end-point of the “EPO in Burns” study regarding the complete re-epithelialization of a defined split skin graft donor site (= the study wound) did not show conclusive results regarding EPO effects on wound healing. Therefore, in this post hoc subgroup analysis, the re-epithelialization of the study wound was evaluated over time using analytic techniques referred to as survival analysis, life tables, and the Cox regression model.

However, several EPO effects on secondary endpoints of the “EPO in Burns” study (safety of EPO treatment in burn patients, SOFA score, and the onset of wound healing) were further investigated. In the previously published main findings of the “EPO in Burns” study, EPO was found to be safe in severely burned patients. In addition, an organ protecting and stabilizing effect in severely burned patients (SOFA score) and an earlier onset of wound healing in the EPO group was demonstrated (Gunter et al., 2018).

In addition, this post hoc subgroup analysis was performed to get a more in-depth view of factors influencing the wound healing process in the severely burned patient focusing on the effects of concomitant medication and gender.

Gender differences in clinical outcomes have been reported in clinical trials (phase I-IV) of different types of disease (Labots et al., 2018). Furthermore, investigating the outcomes of gender differences is now a routine procedure in clinical trials and has become an integral and regulatory factor in the US FDA’s consideration for approval of pharmaceutical products since the mid-1980s (Kessler, 1993). However, gender differences regarding the response to EPO exposure among burn patients have not been studied so far. To date, gender differences regarding morbidity, mortality, and outcomes after burn injuries have only been investigated in a small number of studies. Several of those demonstrated that female burn patients are at a higher risk of fatal outcomes compared to male burn patients (McGwin et al., 2002; Kerby et al., 2006; Karimi et al., 2017), while in another paper young male burn patients had significantly higher mortality rates than young female burn patients (Barrow and Herndon, 1990). Still other studies have reported no statistically significant difference among male and female burn patients (Mostafa et al., 2002; Jeschke et al., 2008; Ederer et al., 2018). Further research is urgently required to investigate possible reasons for this difference between female and male patients. Overall, there is a high demand for improved, safer, and more effective pharmaceutical therapies for men and women suffering from burn injuries.

### Subgroups

The subgroups of this investigation were defined post hoc and not considered in the sample size estimation of the study (power calculation). Therefore, as it is in the nature of a post hoc analysis, statistical hypothesis testing was only exploratory. However, to gain further insight into effects found by the exploratory statistical testing of any post hoc analysis, these results might be used to design new high-quality clinical trials, the results as hypotheses generating tools to develop improved endpoints in the new study’s correct power calculation and to receive statistically significant data.

In our post hoc analysis, the variables defining each subgroup were selected based on two primary considerations: First, the variable in question had to have been discussed in previous research as a potential influence on wound healing. Second, data for each of the resulting subgroups had to be present to permit statistical analysis.

Insulin: The effect of insulin on wound healing is the subject of many studies reporting promising results (Oryan and Alemzadeh, 2017). Improved re-epithelialization of burn wounds has been demonstrated in pre-clinical (Azevedo et al., 2016) and clinical trials (Pierre et al., 1998).

NSAID: Adverse effects of NSAIDs on wound healing are discussed controversially (Stadelmann et al., 1998; Guo and DiPietro, 2010). Discussed mechanisms include reduced collagen metabolism (Klein, 2012), a decrease in the number of fibroblasts in granulation tissue (Krischak et al., 2007), as well as a delay in re-epithelialization and angiogenesis by the inhibition of cyclooxygenase-2 (COX-2) (Futagami et al., 2002).

Vasopressors are indicated for hemodynamic stabilization in severely burned patients (Giessler et al., 2009). It is assumed that an increase of necrotic tissue in burn wounds may be caused by vasopressor-induced vasoconstriction and subsequently decreased perfusion of the tissue, as confirmed in a rabbit burn model (Knabl et al., 1999).

As stated above the subgroups were not prospectively identified, and analyses were only exploratory.

## Materials and Methods

Data for this subgroup analysis were derived from the clinical trial “EPO in Burns,” designed as a prospective, placebo-controlled, randomized, and double-blind trial performed at thirteen study sites throughout Germany. The trial was conducted according to globally accepted standards of good clinical practice in agreement with the Declaration of Helsinki and local regulations. Moreover, it had the full approval of the designated ethics committees of all study sites (leading ethic committee: University of Lübeck, Germany). Informed consent in written form was implemented *via* the “Heidelberger Verfahren” (see also: (Brückner et al., 2010; Gunter et al., 2013)).

The objective of the original clinical trial “EPO in Burns” was to investigate the influence of systemically applied, low-dose recombinant EPO on the wound healing process at a defined split skin graft donor site located at the upper lateral thigh. Therefore, the aim was to get a more in-depth view of the pro-regenerative and cytoprotective effects in thermally injured patients.

Adult patients with full-thickness burn injuries (2b°–3°) were included. Patients received state-of-the-art burn care, including split skin graft transplantation. The study medication, EPO (NeoRecormon^®^, 150 IU per kg body weight, s.c. injection) or a matched placebo (buffered, physiological saline solution), was applied every other day for 21 days. The study wound was defined as a split skin graft donor site (8 cm × 8 cm, 0.3 mm) at a specified location on the upper thigh. According to the trial protocol, standardized wound dressings with polyurethane foil were applied to the study wound. Wound healing stages were monitored clinically and histologically. In addition, laboratory parameters, vital signs, quality of life, scar development, gender differences, and safety parameters including adverse events (AEs) and severe adverse events (SAEs) were investigated.

For further information about the results of the clinical trial “EPO in Burns”, please refer to Gunter et al., 2018. For further information regarding the content of the protocol, the inclusion of unconscious patients by the “Heidelberger-Verfahren” and alternative study methods, please refer to Gunter et al., 2013 (35).

The intention-to-treat (ITT) study population (ITT: n = 84, EPO: n = 45, Placebo: n = 39) of the “EPO in Burns” trial was used for the subgroup analysis in this paper. Thus, patients who received at least one dose of study medication were analyzed according to the treatment they were assigned to by randomization. Subgroups were formed based on gender (female patients: n = 18, EPO n = 12, Placebo n = 6) and concomitant medications: Insulin (Insulin n = 36, EPO n = 17, Placebo n = 19), NASIDs (NASIDs = 41, EPO = 30, Placebo n = 11), and Vasopressors (Vasopressors n = 43, EPO = 20, Placebo n = 23). For a descriptive overview of the characteristics of the study population, see Table 1 in the Results Section.

**TABLE 1:** Baseline characteristics of ITT population.

Baseline characteristics (ITT)			
	EPO (*n* = 45)	Placebo (*n* = 39)	Total (*n* = 84)
Age—years (SD)	48.5 ± 16.2	46.6 ± 15.0	
Age ≥60 years—no. (%)	8 (18%)	10 (26%)	18 (21%)
Female—no. (%)	12 (27%)	6 (15%)	18 (21%)
ABSI Score ≥7—no. (%)	24 (53%)	26 (67%)	34 (40%)
TBS-Sum—% (SD)	24.9 ± 11.7	26.6 ± 13.3	
Death—no. (%)	1 (2%)	1 (2%)	2 (2%)
Subgroups—no. (%)			
Insulin	17 (38%)	19 (49%)	36 (43%)
NASIDs (>1)	30 (67%)	11 (28%)	41 (49%)
Vasopressors	20 (44%)	23 (59%)	43 (51%)

Descriptive statistics are mean and standard deviation or absolute and relative (%) frequency for the ITT population. ABSI score, Abbreviated burn severity index score; The ABSI Score describes the severity of the burn or scald injury and the patient’s prognosis. The lower the ABSI score, the better the patient’s prognosis. An ABSI of over seven relates to a severe threat to life, with a likelihood of survival ranging between 70% and 0% [46]., 1982. TBS-Sum, total body surface thermally injured in %.

### Statistics

The statistical concept we used was the “effect estimation concept”.

Statistical analyses were performed with IBM SPSS Statistics (version 22, IBM Corp., Armonk, N.Y., United States) or SAS (version 9.4.). Subgroup analyses were defined post-hoc, and corresponding hypothesis testing was, therefore, performed on exploratory two-sided 5% significance levels. The distribution of quantitative data is presented using descriptive statistics such as mean, standard deviation, maximum (max), minimum (min), and median, as indicated. Qualitative data are shown as absolute and relative frequencies.

Life table analysis was used to estimate the time-dependent likelihoods of reaching each of the investigated re-epithelialization levels, and the study groups (EPO vs. placebo) were compared using the Wilcoxon tests. Cox proportional hazards regression models were used to assess the interaction between the treatment effect and the factors defining the subgroups (see subgroup description in the introduction of this paper). The models, therefore, included two principal effects for the factor variables study drug and subgroup, as well as the interaction effect of these factors. The Cox model is specified in terms of the hazard. The hazard is the probability that a subject will experience an outcome, here to complete a defined re-epithelialization level of the study wound, in the following unit interval of time given the subject has not yet had the outcome. Instead of interpreting the hazard as a relative risk, we use the term chance. Cox regression models were also used to obtain effect estimates adjusted for gender and the dichotomized ABSI Score (>7 vs. ≤ 7 points).

The designated event (time-to-event outcome) was complete re-epithelialization of the study wound (skin graft donor site) within the observation period (21 days). In addition to complete re-epithelialization (100%), lower levels of re-epithelialization (50% and 75%) were investigated. Therefore, three endpoints given by three levels of re-epithelialization of a defined split skin graft donor site were analyzed. Time-to-event was calculated as the time between day 1 (first administration of the study medication) and the day the individual re-epithelialization level was first achieved. Patients who had not reached any of the defined levels of re-epithelialization by the end of the observation period were treated as censored observations in the analysis.

## Results

### Patient Characteristics and Patient Flow

116 patients were randomized into the trial, 59 (51%) were randomly selected to receive EPO. Of these 116 randomized patients, 84 [45 (54%) EPO, 39 (46%) Placebo] were included in the intention-to-treat population. Overall, 32 patients discontinued study participation upon personal request or at the request of their legal representative: 14 (12%) in the EPO group and 18 (16%) in the placebo group (Gunter et al., 2018).

Age and burn severity (TBS sum) were similar in both treatment arms. However, 24 (53%) patients in the EPO group and 26 (67%) patients in the placebo group showed an ABSI score ≥7 points (cf. Table 1).

### Gender Distribution

There were differences in the gender distribution in the treatment arms (cf. Table 1). Overall, the study included more men, by a factor of almost four (79% men vs. 21% women). In addition, the majority of the women included in the study received EPO [*n* = 12 (27%)], leaving just six women in the placebo group [*n* = 6 (15%)]. Therefore, gender was an additional factor variable to obtain adjusted effect estimates in the Cox proportional hazards models.

### Analysis of Re-Epithelialization Levels

**FIGURE 1:**
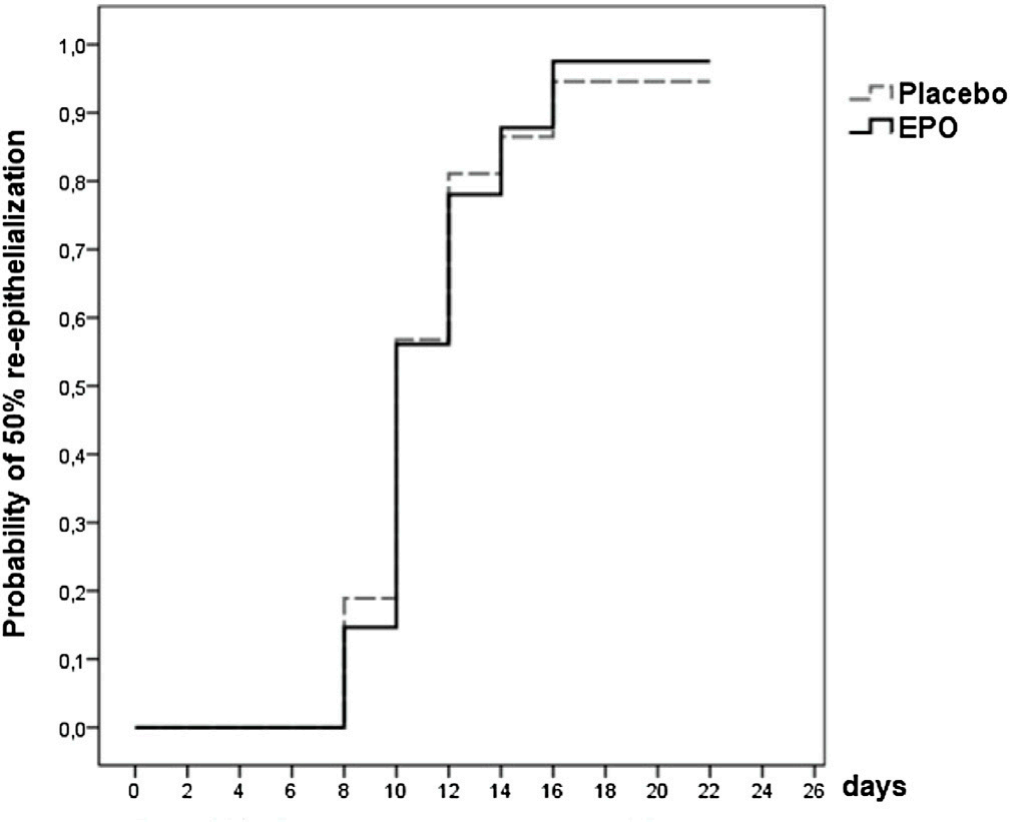
Survival function showing the likelihood of 50% re-epithelialization through day 21 for the patients of the two treatment arms with documentation of the study wound [data were censored for three patients (4%)].

At the end of the observation period, 50% re-epithelialization of the study wound was achieved in a comparable percentage of patients in each treatment group (98% in the EPO group vs. 95% in the placebo group (*p* = 0.802) (cf. Figure 1). The hazard ratio was found to be 1.004, with a confidence interval of (0.638–1.580) (*p* = 0.987) as seen in the median of time needed to reach the 50% re-epithelialization level (cf. Table 2). The number of patients achieving >50% re-epithelialization is shown in Table 3.

**TABLE 2:** Overview of the results of the 50% re-epithelialization level of the study wound.

		EPO (*n* = 41)	Placebo (*n* = 37)	Total (*n* = 78)
50% re-ep*-level	yes	40 (98%)	35 (95%)	75 (96%)
No (%)	no	1 (2%)	2 (4%)	3 (4%)
Median (days)		10	10	
Re-ep*-likelihood		98%	95%	*p* = 0.802

*re-ep, re-epithelialization.

**TABLE 3:** Number of patients achieving over 50% re-epithelialization.

Group	No (%)	Day 06	Day 08	Day 10	Day 12	Day 14	Censored
EPO	(*n* = 41)	6 (15%)	17 (41%)	9 (22%)	4 (10%)	4 (10%)	1 (2%)
Placebo	(*n* = 37)	7 (19%)	14 (38%)	9 (24%)	2 (5%)	3 (8%)	2 (5%)
Total	(*n* = 78)	13 (17%)	31 (40%)	18 (23%)	6 (8%)	7 (9%)	3 (3%)

Looking at the univariable analysis by gender as the only independent variable in the Cox model, the observed differences estimated a hazard ratio of 1.231 [95%-CI: (0.706–2.148), *p* = 0.464] for men vs. women. The interpretation of the hazard ratio suggests the chance (hazard) of achieving >50% re-epithelialization of the study wound of men was increased by 23% compared to the chance of women. In addition, observed differences regarding the univariable analysis of the ABSI score (ABSI ≥7) showed only a negligible difference in achieving the 50% level of re-epithelialization [hazard ratio: 0.971, 95%-CI: (0.606–1.557), *p* = 0.902].

**FIGURE 2:**
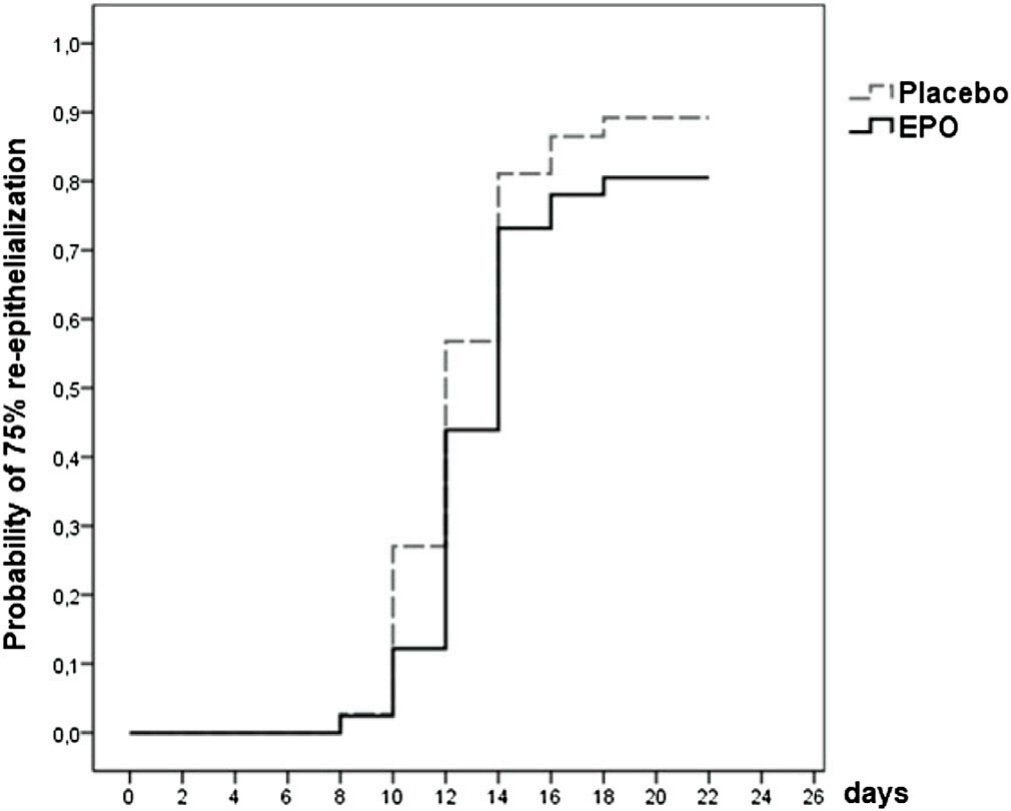
Survival function showing the likelihood of 75% re-epithelialization through day 21 for patients from both treatment arms whose study wound healing process was fully documented [data were censored for 12 (15%) patients].

Of the 78 patients whose study wound healing process was fully documented, 66 (85%) reached a re-epithelialization of over 75% within the observation period. Of these 66 patients, 33 were part of the EPO group, representing 80%. The remaining 33 patients in the placebo group made up 89% (cf. Table 4 and Figure 2).

**TABLE 4:** Overview of the results of the 75% re-epithelialization level of the study wound.

		EPO (*n* = 41)	Placebo (*n* = 37)	Total (*n* = 78)
75% re-ep*-level	yes	33 (80%)	33 (89%)	66 (85%)
No (%)	no	8 (20%)	4 (11%)	12 (15%)
Median (days)		14	12	
Re-ep*-likelihood		80%	89%	*p* = 0.132

*re-ep, re-epithelialization.

The hazard ratio for reaching the 75% level of re-epithelialization was 0.749 [95%-CI: (0.462–1.214), *p* = 0.241]. Therefore, interpretation of the hazard ratio indicates a 25% lower chance of reaching this level within the observed time for the EPO group compared to the placebo group.

Table 5 lists the number of patients that achieved over 75% of re-epithelialization on the designated day.

**TABLE 5:** Number of patients achieving over 75% re-epithelialization.

Group	No (%)	Day 06	Day 08	Day 10	Day 12	Day 14	Day 16	Censored
EPO	(*n* = 41)	1 (2%)	4 (10%)	13 (32%)	12 (29%)	2 (5%)	1 (2%)	8 (20%)
Placebo	(*n* = 37)	1 (3%)	9 (24%)	11 (30%)	9 (24%)	2 (5%)	1 (3%)	4 (11%)
Total	(*n* = 78)	2 (3%)	13 (17%)	24 (31%)	21 (27%)	4 (5%)	2 (3%)	12 (14%)

The hazard ratio was 1.240 [95%-CI: (0.676–2.276), *p* = 0.487] in a univariable analysis by gender comparing men to women. The interpretation of the hazard ratio suggest men seem to have a 24% higher chance of reaching the 75% reepithelialization level. Observed differences looking at the univariable hazard ratio of the ABSI score ≥7 which was 0.803 [95%-CI: (0.488–1.321), *p* = 0.388], would suggest a 20% lower chance of reaching over 75% re-epithelialization for patients with an ABSI score ≥7 compared to patients with an ABSI score of six points or less.

At the end of the observation time, complete (100%) re-epithelialization of the study wound was reached in 22 (28%) of the 78 patients analyzed. Of these 22 patients 11 (27%) received EPO and 11 (30%) received placebo (cf. Table 6).

**TABLE 6:** Overview of the results of the 100% reepithelialization level of the study wound.

		EPO (*n* = 41)	Placebo (*n* = 37)	Total (*n* = 78)
100% reep*-level	yes	11 (27%)	11 (30%)	22 (28%)
No (%)	no	30 (73%)	26 (70%)	56 (72%)
Median (days)		21, 0	21, 0	
Reep*-likelihood		27%	30%	*p* = 0.965

*reep, reepithelialization The median time to complete reepithelialization is not reached.

**FIGURE 3:**
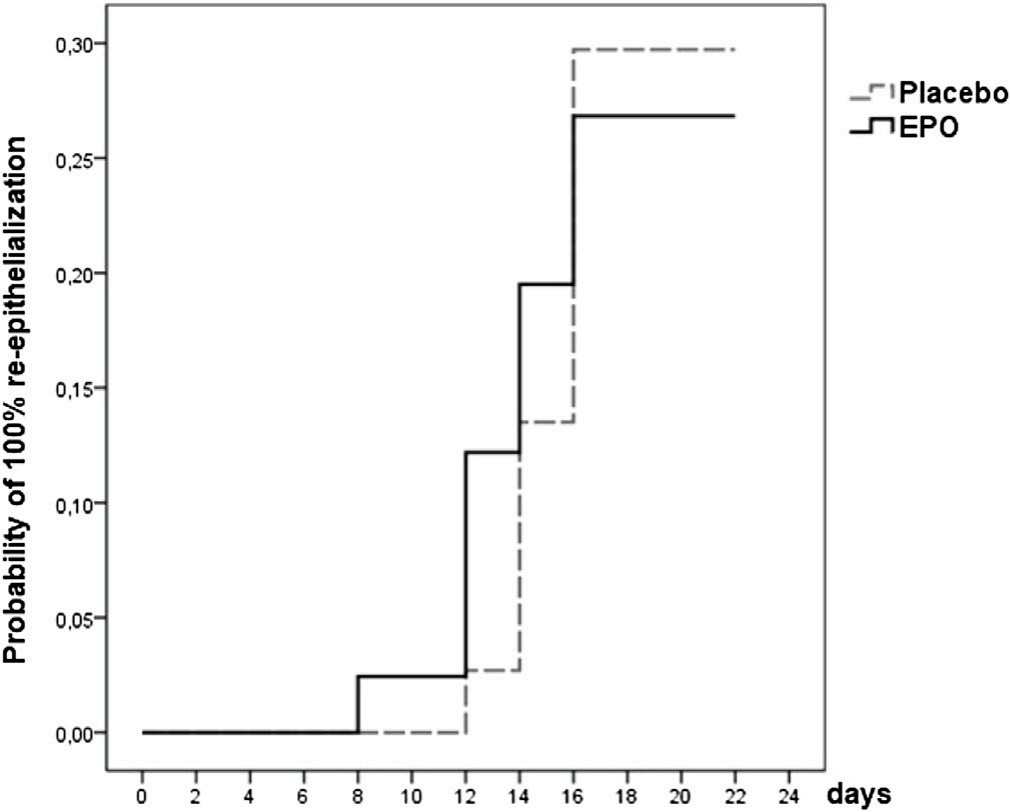
Survival function showing the likelihood of 100% re-epithelialization through day 21 for patients from both treatment arms whose study wound healing process was fully documented (data were censored for 56 (72%) patients).

The likelihood of reaching complete re-epithelialization in the two treatment arms by day 21 is pictured in the survival function graph (cf. Figure 3). There was a slight difference regarding the number of patients who reached this level in the respective treatment arms (27% in the EPO group vs. 30% in the placebo group (*p* = 0.965), hazard ratio: 0.966, 95%-CI: (0.419–2.227); *p* = 0.935). Table 7 lists the number of patients achieving complete re-epithelialization on the designated day.

**TABLE 7:** Number of patients achieving complete re-epithelialization.

Group	No	Day 06	Day 08	Day 10	Day 12	Day 14	Censored
EPO	(*n* = 41)	1 (3%)	0	4 (10%)	3 (7%)	3 (7%)	30 (73%)
Placebo	(*n* = 37)	0	0	1 (3%)	4 (11%)	6 (16%)	26 (70%)
Total	(*n* = 78)	1 (1%)	0	5 (6%)	7 (9%)	9 (12%)	56 (72%)

In the Cox model, the hazard ratio (univariable analysis) adjusted for gender was 5.984 [95%-CI: (0.805–44.490), *p* = 0.080]. These findings seem to indicate that men have a 6 times higher chance of reaching 100% re-epithelialization.

The interpretation of the hazard ratio suggests an ABSI score ≥7 as a variable hazard ratio: 0.330, 95% CI: (0.141–0.774), (*p* = 0.011). This means that an ABSI score ≥7 lowers the chance of patients reaching 100% re-epithelialization by 67%.

### Subgroup Analysis of Respective Concomitant Medication

Please note that we have used the “effect estimation concept” as the statistical model for this paper.

Insulin: Out of 84 patients, 36 (43%) received insulin. Table 8 provides an overview of this subgroup’ results.

**TABLE 8:** Likelihood of achieving the designated re-epithelialization levels within the observation period by subgroup stratum.

Re-ep*-level	Insulin		No insulin		Total
	EPO + I	Placebo + I	EPO	Placebo	
%, (no)	(*n* = 17)	(*n* = 19)	(*n* = 24)	(*n* = 18)	(*n* = 78)
50%	100% (17)	95% (18)	96% (23)	94% (17)	96% (75)
75%	82% (14)	84% (16)	79% (19)	94% (17)	85% (66)
100%	6% (1)	16% (3)	42% (10)	44% (8)	28% (22)

* re-ep, re-epithelialization; 36 of 84 patients received insulin, insulin group: EPO + insulin (E + I), placebo + insulin (P + I), no insulin group: EPO (E), placebo (P).

50% re-epithelialization level: In the Cox model, the observed hazard ratio for reaching this level in the insulin group was 1.155 [95%-CI: (0.592–2.251)], in the group receiving no insulin, the ratio was 0.892 [95%-CI: (0.475–1.673)]. The interaction between the insulin group and the study group suggested a hazard ratio of 1.307 [95%-CI: (0.521–3.277), *p* = 0.568].

75% re-epithelialization level: The observed therapy effect of EPO was negative in both subgroups [insulin: hazard ratio: 0.815, 95%-CI: (0.397–1.672); in the no insulin group: hazard ratio: 0.686, 95%-CI: (0.356–1.323)]. The interaction term resulted in a hazard ratio of 1.199 [95%-CI: (0.453–3.173), *p* = 0.715]; the EPO’s therapeutic effect in the insulin subgroup suggested superior results by a factor of 1.199.

100% re-epithelialization level: For patients who did not receive insulin, the therapeutic effect of EPO seemed to be positive [hazard ratio: 1.070, 95%-CI: (0.422–2.711)]. The hazard ratio in the insulin group was 0.368 [95%-CI: (0.038–3,536)]. The interaction between the insulin group and the study group suggested a reduction in the therapeutic effect of EPO [hazard ratio: 0.341, 95%-CI: (0.030–3.937), *p* = 0.389].

**FIGURE 4:**
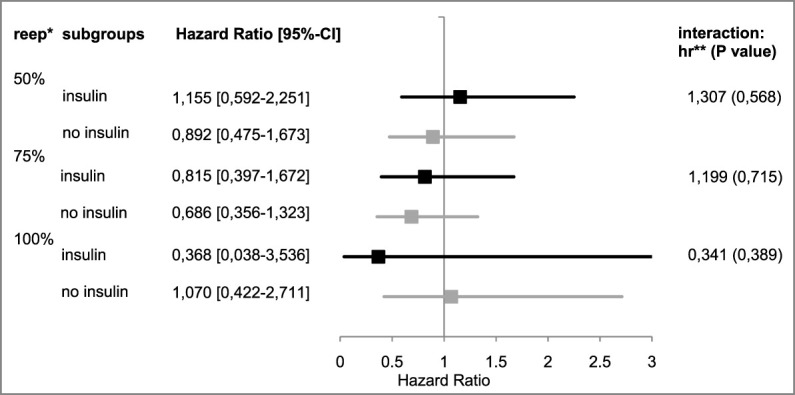
Hazard ratios of “insulin” vs. “no insulin” subgroups. *re-ep, re-epithelialization level; ** hr, hazard ratio; represents the therapeutic effect of EPO with its 95%¬ confidence interval in the subgroup; and hr < 1 represents a negative therapeutic effect in the patients receiving EPO; the respective interaction effects are listed on the right.

Figure 4 provides an overview of the Cox regression model results for all levels of re-epithelialization.

A difference between patients receiving or not receiving insulin, respectively, was observed in the median time required to reach the respective re-epithelialization levels (cf. Table 9).

**TABLE 9:** Median time required to reach the designated re-epithelialization level in each subgroup stratum in days.

Median (days)	EPO + Insulin	Placebo + Insulin	EPO	Placebo
50%	9.38	9.50	10.00	9.75
75%	12.20	11.50	12.57	11.60
100%	21.00	21.00	21.00	21.00

**FIGURE 5:**
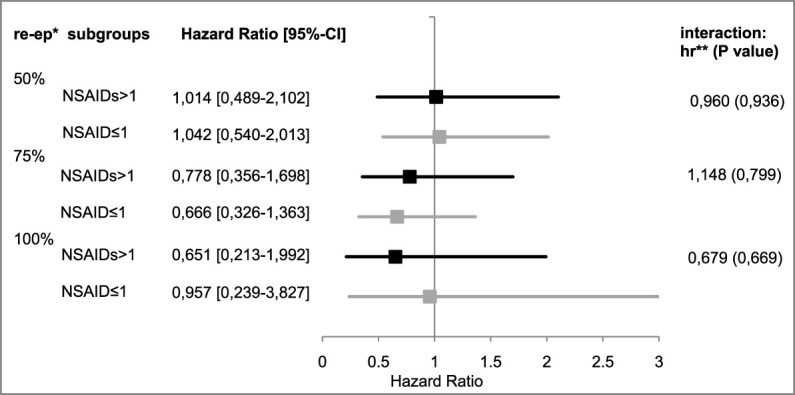
Hazard ratios of subgroup “NSARs >1” vs. “NSAIDs ≤1”. *re-ep, re-epithelialization level; **hr, hazard ratio; represents the therapeutic effect of EPO with its 95%¬ confidence interval in the subgroup, a hr < 1 represents a negative therapeutic effect for the patients who received EPO; the interaction effects are listed on the right.

Non-steroidal anti-inflammatory drugs (NSAIDs): 41 (49%) of the 84 patients received more than one non-steroidal anti-inflammatory drug. Table 10 and Figure 5 provide an overview of the results of this subgroup analysis.

**TABLE 10:** Likelihood of achieving the designated re-epithelialization levels within the observation period by subgroup stratum.

Re-ep*-level	NSAIDs > 1		NSAID ≤ 1		Total
	EPO	Placebo	EPO	Placebo	
	(*n* = 27)	(*n* = 10)	(*n* = 14)	(*n* = 27)	(*n* = 78)
50%	96% (26)	100% (10)	100% (14)	93% (25)	96% (75)
75%	81% (22)	90% (9)	79% (11)	89% (24)	85% (66)
100%	30% (8)	50% (5)	21% (3)	22% (6)	28% (22)

* re-ep = re-epithelialization; 41 of 84 patients received more than one NSAID, NSAIDs > 1 group: EPO + NSAIDs > 1 (E + NSAIDs > 1), placebo + NSAIDs > 1 (P + NSAIDs > 1), NSAID ≤ 1 group: EPO (E), placebo (P).

50% re-epithelialization level: The observed hazard ratio for reaching the 50% level of re-epithelialization was slightly above 1 in both subgroup strata [NSAIDs>1: hazard ratio: 1.014, 95%-CI: (0.489–2.102); NSAIDs ≤ 1: hazard ratio: 1.042, 95%-CI: (0.540–2.013)].

75% re-epithelialization level: The observed hazard ratio for reaching the 75% level of re-epithelialization was less than 1 in both subgroup strata [NSAIDs>1: hazard ratio: 0.779, 95%-CI: (0.356–1.698); NSAIDs ≤ 1: hazard ratio: 0.666, 95%-CI: (0.326–1.363)]. This seems to correspond to the estimate of the EPO’s therapeutic effect on the overall cohort.

100% re-epithelialization level: The observed hazard ratio in the stratum NSAIDs ≤ 1 was 0.957 [95%-CI: (0.239–3.827)]. The hazard ratio in the stratum NSAIDs>1 was 0.651 [95%-CI (0.213–1,992)], indicating a lower chance of reaching this level for patients receiving EPO. The observed hazard ratio of the interaction term, therefore, seemed to be less than 1 [hazard ratio: 0.679, 95%-CI: (0.114–4.025), *p* = 0.669].

Vasopressors (VP): 43 (51%) of all 84 randomized patients received vasopressors. 35 of these patients showed an ABSI score ≥7 (total: 35/43 (81%). In the EPO group 14/35 (40%) received vasopressors. In the Placebo group: 21/35 (60%) received vasopressors. 41 (49%) patients received no vasopressors.

Table 11 provides an overview of the number of patients in the four subgroup strata.

**TABLE 11:** Likelihood of achieving the designated re-epithelialization levels within the observation period by subgroup stratum.

Re-ep*-level	Vasopressors (VP)		No vasopressors		Total
	EPO + VP	Placebo + VP	EPO	Placebo	
	(*n* = 18)	(*n* = 21)	(*n* = 23)	(*n* = 16)	(*n* = 78)
50%	94% (17)	95% (20)	100% (23)	94% (15)	96% (75)
75%	83% (15)	86% (18)	78% (18)	94% (15)	85% (66)
100%	22% (4)	19% (4)	30% (7)	44 (7)	28% (22)

*re-ep, re-epithelialization; 43 of 84 patients received vasopressors, vasopressor group: EPO + vasopressors (E + VP), placebo + vasopressors (P + VP), no vasopressors group: EPO (E), placebo (P).

50% re-epithelialization level: The observed hazard ratio in the subgroup stratum “VP” was 0.901 [95%-CI: (0.471–1.723)]. For patients who did not receive vasopressors, the observed hazard ratio was 1.062 (95%-CI: (0.554–2.035]). The interaction effect was calculated with a hazard ratio of 0.855 (95%-CI: (0.342–2.143], *p* = 0.739).

75% re-epithelialization level: The interaction term of the EPO’s therapeutic effect in this subgroup analysis seemed to be positive with an observed hazard ratio of 1.403 [95%-CI: (0.531–3.708), *p* = 0.495], but was found to be negative in both strata [VP: hazard ratio: 0.870, 95%-CI: (0.438–1.729); no VP: hazard ratio: 0.623, 95%-CI: (0.313–1.240)].

**FIGURE 6:**
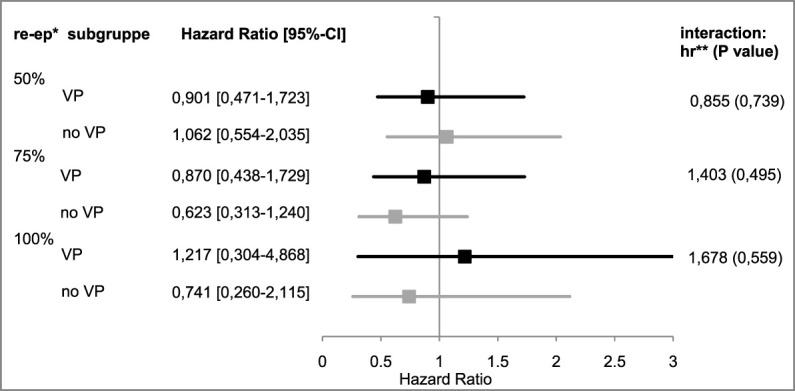
Hazard ratios of subgroup “VP” vs. “no VP”. *re-ep = re-epithelialization level; **hr = hazard ratio; represents the therapeutic effect of EPO with its 95%¬ confidence interval in the subgroup, an hr < 1 represents a negative therapeutic effect for the patients who received EPO. The interaction effects are listed on the right by the hr, including *p*-values.

100% re-epithelialization level: With an observed hazard ratio of 1.217 [95%-CI: (0.304–4.868)], the therapeutic effect of EPO for the VP stratum was positive. The interaction effect seemed to be greater by a factor of 1.678 [95%-CI: (0.295–9.546), *p* = 0.559]. In the no VP stratum, the observed hazard ratio was 0.741 [95%-CI: (0.260–2.115)] (see Figure 6).

The results of the Cox regression are summarized in Figure 3.

**FIGURE 7:**
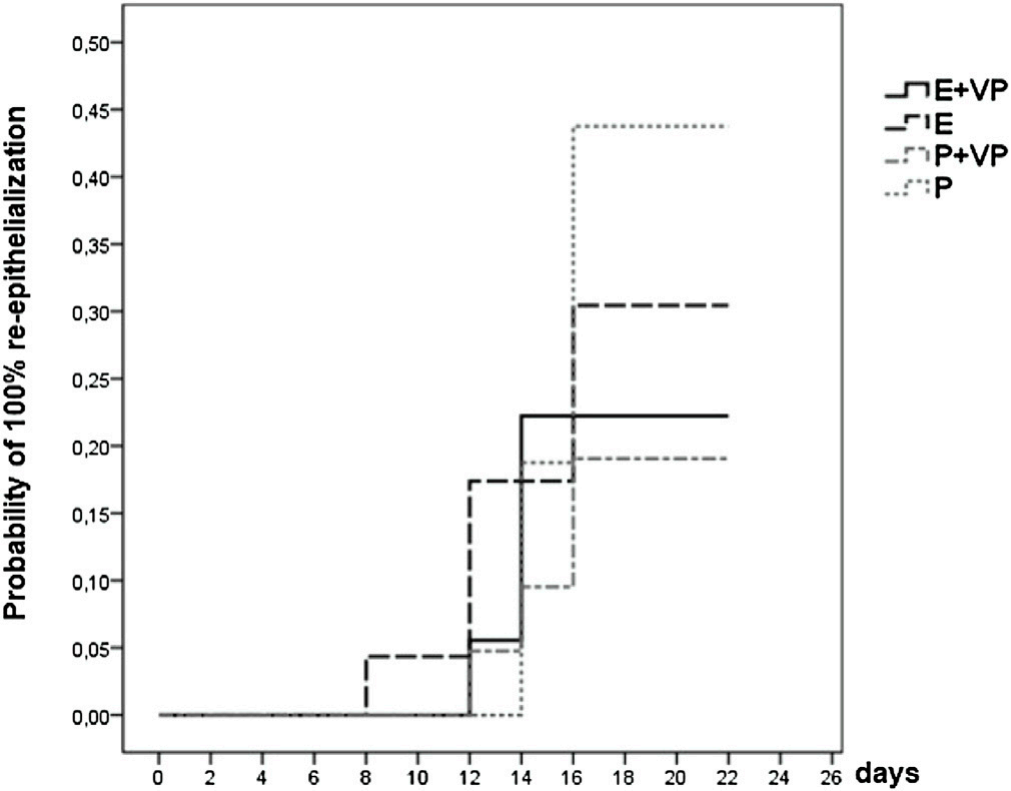
Survival function of subgroup “VP” vs. “no VP”. Showing the likelihood of 100% re-epithelialization through day 21 for patients from four subgroup strata whose study wound healing process was fully documented (data were censored for 56 (72%) patients).

As seen in Figure 7, at the end of the observation period, complete re-epithelialization of the study wound was reached in 30% of patients receiving only EPO (E) (no vasopressors), and in 44% of patients receiving only the placebo (P) (no vasopressors). Patients receiving only Placebo or only EPO showed a greater likelihood of healing completely than patients in the subgroup stratum vasopressors = “VP” (EPO + VP and Placebo + VP).

## Discussion

The results described in this post hoc analysis are based on subgroup data collected from the randomized, placebo-controlled clinical trial “EPO in Burns”, which investigated the regenerative effects of low-dose recombinant EPO on split skin graft donor sites in severely burn-injured patients.

Subgroup analysis revealed that the trend for the survival function of the 50% re-epithelialization level was approximately similar for both treatment arms. A higher likelihood of reaching the 75% re-epithelialization level of the study wound within the observation period was recorded for the placebo group. The median time by which this re-epithelialization level was reached in the EPO group was 1 day later. During the first 10 days of observation, the EPO group demonstrated a higher likelihood of reaching 100% re-epithelialization. The placebo group only showed a higher likelihood of complete re-epithelialization after this initial 10-day period. As described in the publication by (Gunter et al., 2018), patients of the EPO group showed a significant increase in serum EPO levels during the first 10 days after the onset of therapy, which decreased without changes in the dosing regimen after day 10. Several publications postulated that EPO can only overcome the inhibitory effect of pro-inflammatory cytokines with high serum concentrations (1–20 nmol/L) since the EPO-hetero receptor has been proposed to have a lower affinity compared to the EPO-homo (EPOR_2_) receptor (Brines and Cerami, 2008). Therefore, EPO might only develop its pro-regenerative effects, including its tissue-protective effects, in higher concentrations.

The trend towards an early positive effect of EPO during the wound healing process followed by a contrary impact over time has previously been described in animal studies. It is discussed in connection with the selected dosage, the duration of the application, and the time of the EPO application. Saray et al. (Saray et al., 2003) described EPO’s impact as being dependent on the duration of the application. EPO administration over a short period of time (single shot or up to 5 days) resulted in a better flap survival compared to EPO application over 3 weeks (Saray et al., 2003). Sorg et al. (2009) showed that a single, high-dosage (5000 IU) shot of EPO improved re-epithelialization and induced a timely vascular maturation. Repetitive administration of high-dose EPO, on the other hand, impaired the healing process, resulting in delayed re-epithelialization and vascular refinement (Sorg et al., 2009). Further preclinical studies support this hypothesis. Rezaeian et al. discussed that an application performed over 3 days but not exceeding an application period of 10 days could contribute to a faster re-epithelialization of the experimental wounds (Rezaeian et al., 2008). During this period, EPO unfolds its anti-apoptotic, anti-inflammatory, and pro-antiangiogenic effects. Arslantas et al. describe an application time of 5 days after setting a trauma as optimal (Arslantas et al., 2015).

The selection of dosing and timing of EPO-administration in the “EPO in Burns” trial was based on selected schemes of human studies on the cytoprotective effect of EPO (Ehrenreich et al., 2002; Keast and Fraser, 2004). In addition, knowledge on pharmacokinetics and pharmacodynamics gained from studies in dialysis patients (Cheng et al., 1991; Lui et al., 1991) and severely ill patients (Corwin et al., 2007) was used, as well as the manufacturer’s instructions (Roche Pharma, 2015). Discussing the results critically, neither the chosen dosing nor the application scheme seemed to have been optimal.

The phenomenon that EPO has relevant protective effects in animal studies that cannot be reproduced in human studies has also been described as the “EPO-paradox” (Steppich et al., 2017). As an explanation for this paradox, the various dosage regimes of EPO-administration, the onset of therapy after trauma, and the period of time of application have been discussed.

Another explanation for the “EPO-paradox” may be the limited transferability of results from preclinical studies of mostly healthy animals to often seriously ill patients in clinical studies (Solling, 2012). Differences in the skin of animal models compared to humans have also been discussed. Human skin is thicker than, for example, rat skin (Tobalem et al., 2013). Another apparent difference to human skin is the higher density of hair. Hair follicles are rich in stem cells. Thus, processes of mobilization of stem cells and hair growth seen in rodents can only partially be expected to be observed in humans (Abdullahi et al., 2014; Eming et al., 2014). Investigations into the pharmacokinetics and pharmacodynamics of EPO in different species suggest a better distribution of EPO in smaller animals (Woo and Jusko, 2007). This might be another reason for the improved effectiveness of EPO in animal models.

A new finding of the present study is an apparent unfavorable trend in the wound healing process of women, regardless of the study medication. The reason for a possible female disadvantage in wound healing remains unclear. No clinical studies investigating differences in the healing of burn wounds related to gender could be found. In terms of mortality, women have a higher risk of dying from burns. This is the reason why in the ABSI score, proposed by Tobiasen et al., in 1982 (Tobiasen et al., 1982) women receive a score for their gender, denoting a worse prognosis compared to men. Retrospective studies conducted in the early 2000s in the United States (Kerby et al., 2006; McGwin et al., 2002; O'Keefe and Hunt, 2001), as well as a prospective study undertaken in Australia and New Zealand (Moore et al., 2014) confirmed and reconfirmed this finding. The increased mortality among female patients was independent of the extent (TBS) and the depth of their burn wound. The authors discussed a lower muscle mass as an explanation for the increased mortality of female burn victims, as it might result in increased fluid loss and, therefore, a greater risk of infection. Moreover, delayed immune response and higher estrogen levels were suggested as possible causes, but none of these assumptions has been investigated and confirmed in clinical trials. In general, one can say that few studies have focused on gender-specific differences in burn medicine (Pauzenberger et al., 2017).

Insulin is the gold standard in the treatment of hyper-metabolism after burns. It improves the outcome of severely burnt patients and reduces the incidence of infections, SIRS, and sepsis (Jeschke et al., 2016). A positive effect of insulin on wound healing in severely burnt patients has been shown in several studies (Pierre et al., 1998; Tuvdendorj et al., 2011; Wang et al., 2016).

As described in the subgroup results for insulin, overlaps with EPO’s mechanisms of action on wound healing might exist. Insulin partially activates the same intracellular signaling pathways as EPO (Oryan and Alemzadeh, 2017), leading to the assumption that the simultaneous use of EPO and insulin may cause a possible amplification of the mentioned processes during early wound healing. In addition, EPO promotes insulin sensitivity, as evidenced by experimental studies (Niu et al., 2016) and studies in dialysis patients (Mak, 1996; Mak, 1998).

An augmented effect of insulin, both systemically and on the wound healing process, might, therefore, be achieved *via* the simultaneous administration of EPO. The combination of these two drugs during the early stages of wound healing might lead to an exciting new option for innovative burn wound therapy in the future.

In this subgroup analysis, no apparent negative influence on wound healing was revealed by the application of one or more than one NSAID. On the contrary, the NSAIDs>1 subgroup (E + NSAIDs>1, P + NSAIDs>1) had a higher likelihood of reaching the 100% re-epithelialization level. Patients who received the placebo appeared to be at an advantage.

In the literature, the use of NSAIDs in the healing process of soft tissue has been discussed controversially. Due to their desired anti-inflammatory properties as pain medications, they are credited with an anti-proliferative effect (Guo and DiPietro, 2010). Explanations for this effect are, among others, the inhibition of the enzymes COX1 and COX2, followed by decreased prostaglandin synthesis (Chen and Dragoo, 2013). COX2 has been described as essential in the healing of fractures (Simon et al., 2002) and the re-epithelialization of wounds (Futagami et al., 2002). In this field of research, however, the lack of clinical studies makes it impossible to provide a definitive answer (Krischak et al., 2007; Chen and Dragoo, 2013).

Subgroup data point to a hemodynamically stabilizing influence of EPO, in line with its previously demonstrated effect as a vasopressor. These attributes can be beneficial to patients in the treatment of sepsis or shock (Walden et al., 2010). On the other hand, by the same reasoning, these same effects of EPO have previously been blamed for the occurrence of arterial hypertension (Vaziri and Zhou, 2009). In the present study, 43 (51%) patients received vasopressors during the investigational period. This fact illustrates the need for hemodynamically stabilizing drugs in cases of severe burn injuries. An interesting finding was that vasopressors were required less often in the EPO group (EPO: 44%; Placebo: 59%). EPO may have a positive effect on hemodynamically unstable patients. This corresponds to reduced morbidity and a better prognosis for patients receiving EPO, as described by Gunter et al. (Gunter et al., 2013). Another clinical study supporting the observation of a circulation stabilizing effect of EPO is the publication of Corwin et al. (Corwin et al., 2007). The authors describe reduced morbidity and mortality in severely ill patients under EPO therapy.

These hemodynamically stabilizing effects of EPO seem to be independent of its erythropoietic effects. In the “EPO in Burns” study (Gunter et al., 2013), patients receiving EPO did not show increased hematocrit and hemoglobin levels compared to the placebo group, suggesting that EPO can achieve hemodynamically stabilizing effects without changing the blood rheology (Krapf and Hulter, 2009).

Data from the subgroup of patients receiving vasopressors were less likely to reach 100% re-epithelialization (hr = 0.551). However, 81% of these patients showed an ABSI score ≥7. An ABSI score ≥7 indicates a severe threat of life (with a probability of survival of 50–70%) (Tobiasen et al., 1982), implying that these patients are in critical clinical condition. In addition, “total body surface area burned” is an important single score within the ABSI score based on the empirical observation that higher numbers cause delays in the wound healing process. A delayed wound healing process, in turn, increases the risk of a fatal outcome. Moreover, it is assumed that increased necrotic tissue in burn wounds may be caused by vasopressor-induced vasoconstriction and the subsequently decreased perfusion of the tissue, as confirmed in a rabbit burn model (Knabl et al., 1999).

### Limitations

Our results were generated by post hoc subgroup analyses of a limited number of patient data documented in 116 recruited patients. Therefore, no statistical significance can be generated.

Due to the explorative character of the analysis uncontrolled confounding can occure. Likewise, the presented univariable analyses provide marginal effect estimates without adjustment for confounding.

To reduce bias diversity of recruited study patients should be minimized. The chosen inclusion- and exclusion criteria of the clinical trial helped to minimize patient diversity. However, with real patients standardization is limited.

## Conclusion

A noteworthy finding of the post hoc subgroup analysis was that women demonstrated a lower chance of reaching the same wound healing levels within a certain period of time compared to men, irrespective of their treatment group.

Subgroup analyses further revealed that patients treated with EPO had a higher chance of reaching 100% re-epithelialization within the first 10 days. If such an effect is substantiated, this finding suggests new therapeutic options.

Finally, analysis of the subgroup of patients receiving vasopressors revealed a trend of decreased need for vasopressors in patients receiving EPO. This may indicate a hemodynamically stabilizing effect of EPO in severely burnt patients that could point to new therapeutic options.

In summary, the results of this post hoc subgroup analysis provide a starting point for further preclinical and clinical investigations to gain a deeper understanding of the underlying mechanisms of the revealed effects.

## EPO in Burns Study Group

Dornseifer U, Dunda S, Ernert C, Grieb G, Hartmann B, Lonic D, Mailänder P, Namdar T, Neugebauer E, Ninkovic M, Ohmann C, Otte M, Ottomann C, Pallua N, Pierson T, Reichert B, Ryu S-M, Schaller H. E, Siemers F, Sievers R, Steen M, Thamm O. C, Von Wild T, Wolter T.

## Data Availability

The original contributions presented in the study are included in the article/Supplementary Material, further inquiries can be directed to the corresponding author.
